# Supplementary material for: Genetic Editing of Tomato Golgi-Localized Nucleotide Sugar Transporter 1.1 Promotes Immunity Against Phytophthora infestans
Source: Genes (Basel). 2025 Apr 21;16(4):470. doi: 10.3390/genes16040470 (PMC12026973; doi:10.3390/genes16040470)
Supplement: Supplementary file 1 [file genes-16-00470-s001.zip › Supplemental Figure.pdf]

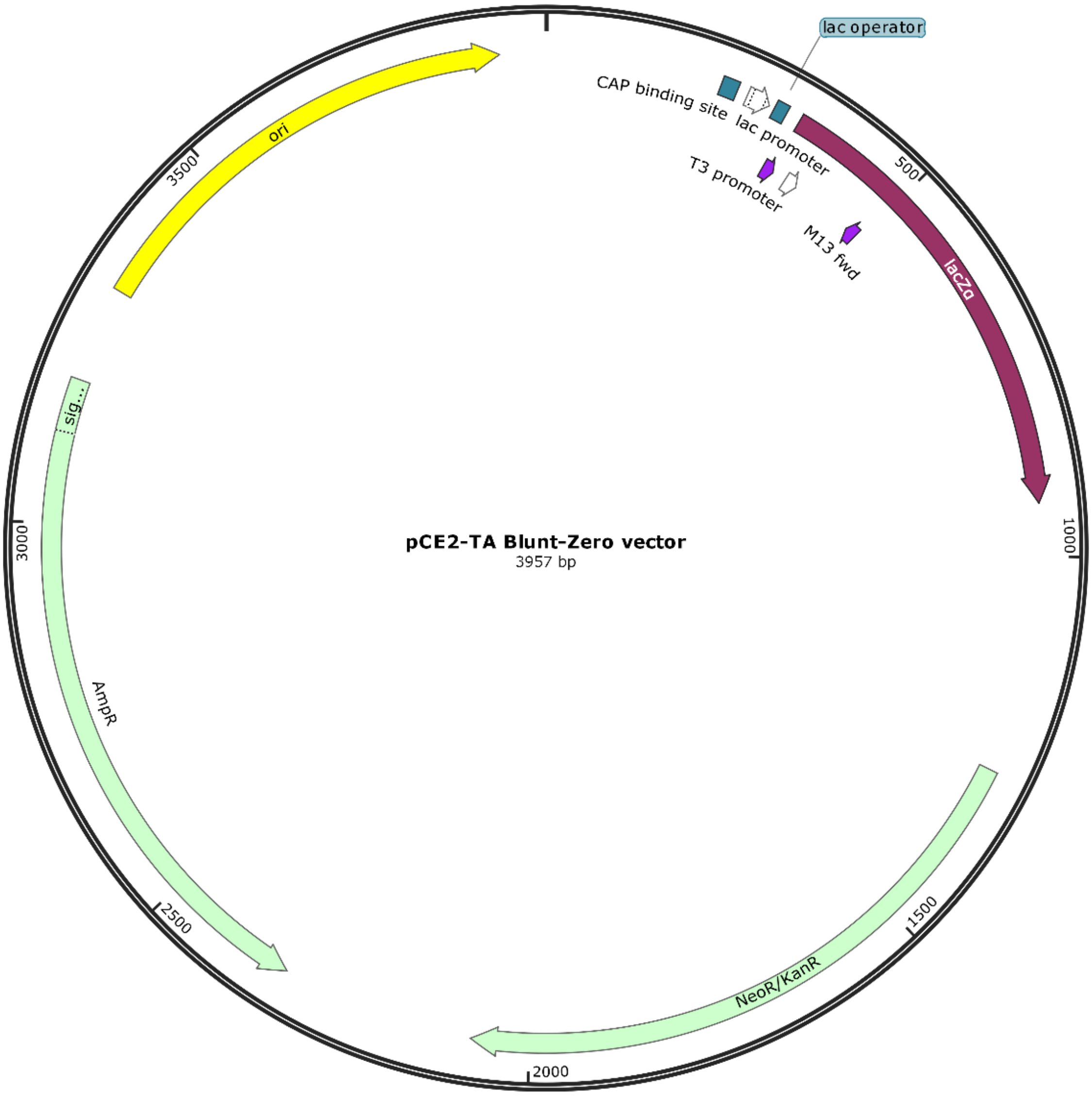

Supplemental Figure 1. The vector map information of pCE2-TA-Blunt-Zero_vector

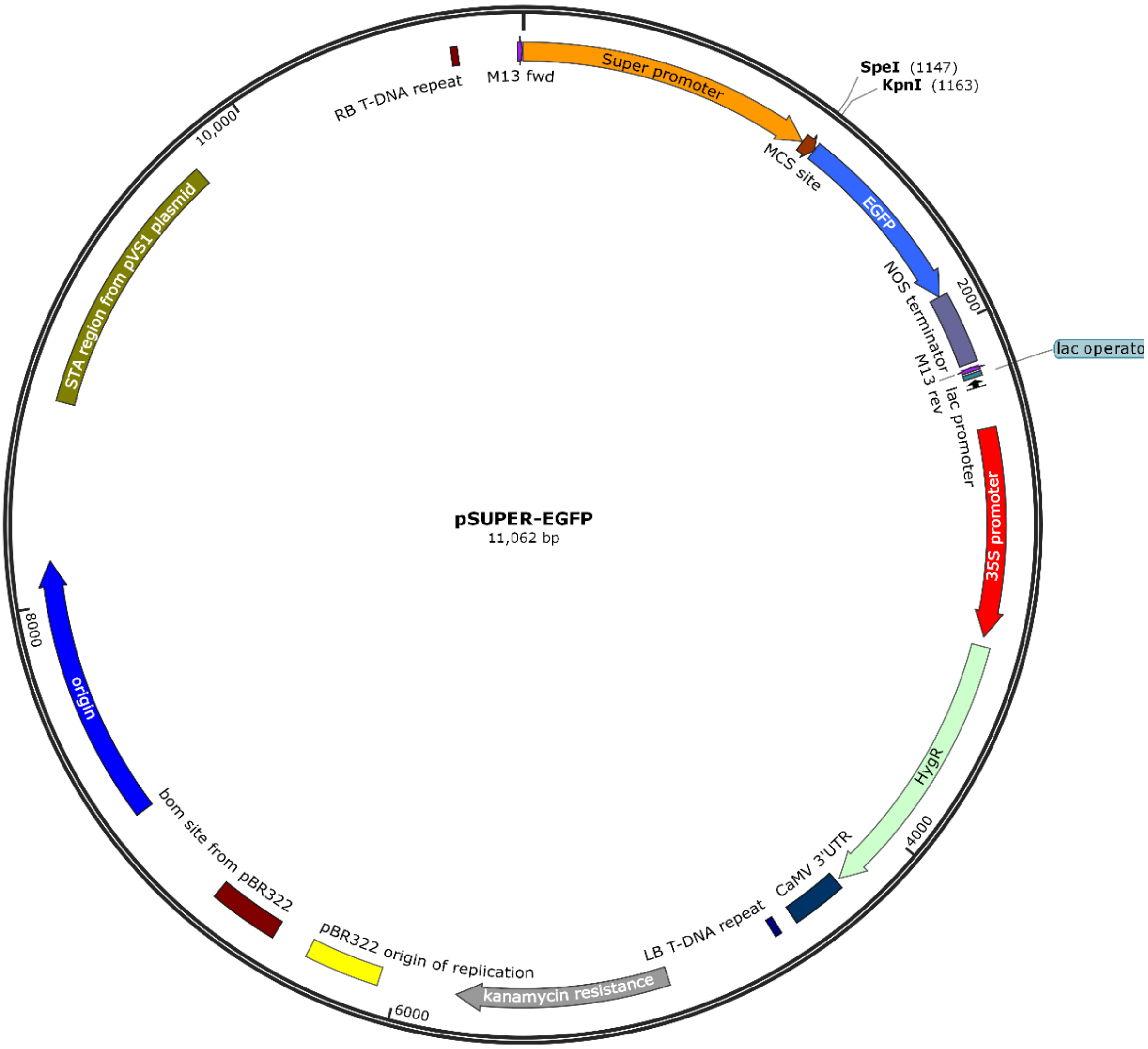

Supplemental Figure 2. The vector map information of pSUPER-EGFP

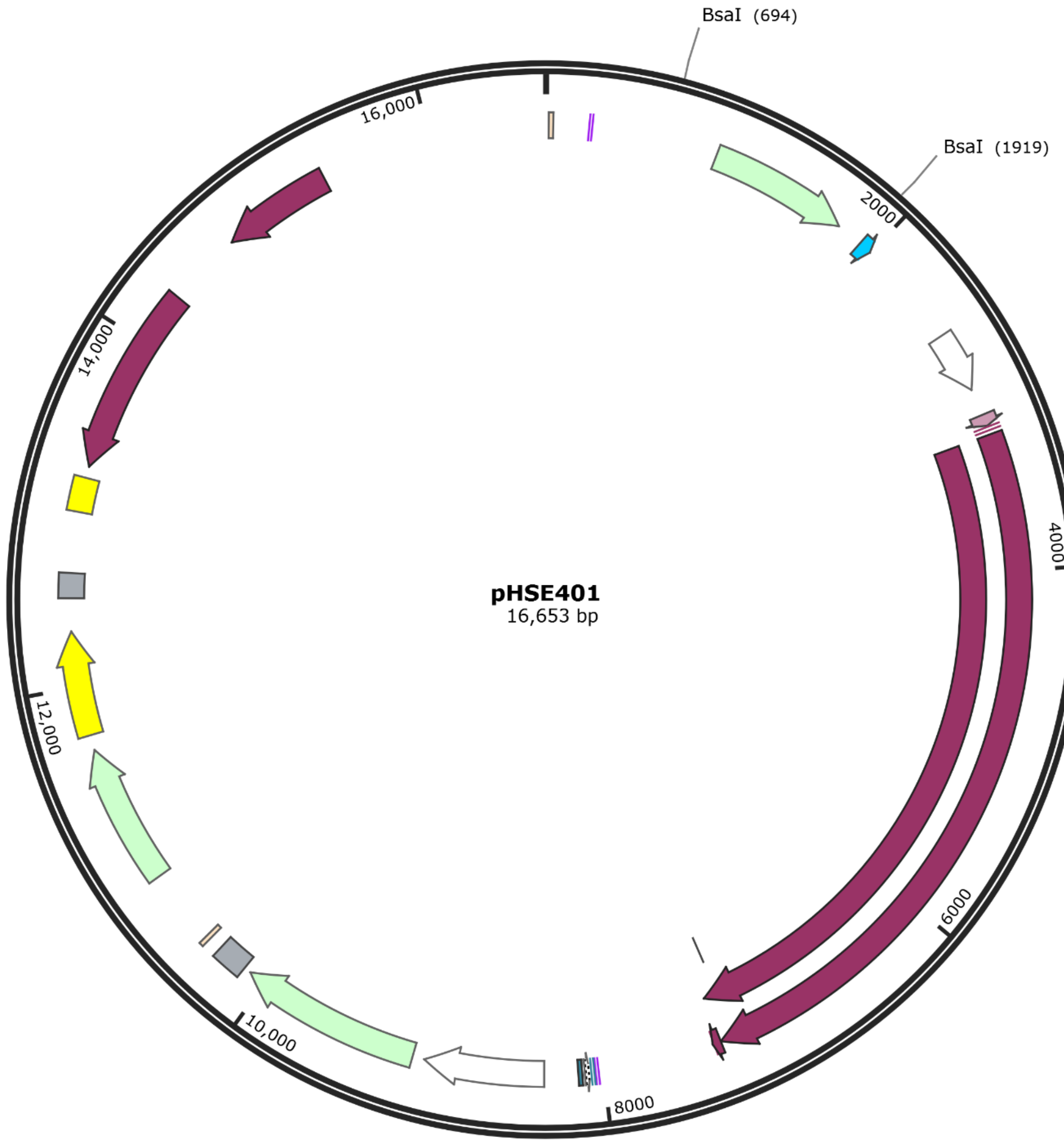

Supplemental Figure 3. The vector map information of pHSE401
